# Integrative Analysis of Metabolome and Transcriptome Reveals Molecular Insight into Metabolomic Variations during Hawthorn Fruit Development

**DOI:** 10.3390/metabo13030423

**Published:** 2023-03-13

**Authors:** Yan Wang, Ruixin Hao, Rongkun Guo, Huilan Nong, Yu Qin, Ningguang Dong

**Affiliations:** Institute of Forestry and Pomology, Beijing Academy of Agriculture and Forestry Sciences, Beijing Engineering Research Center for Deciduous Fruit Trees, Beijing 100093, China; wangyanifp@baafs.net.cn (Y.W.); haoruixinemail@gmail.com (R.H.); guorongkunemail@gmail.com (R.G.); lanlanlan0108@gmail.com (H.N.); qinyuemail@gmail.com (Y.Q.)

**Keywords:** broadly targeted metabolome, metabolites, fruit development and ripening, flavonoids, hawthorn, transcriptome

## Abstract

Hawthorn (*Crataegus pinnatifida* var. *major*), a cultivated fruit tree, is native and unique to China. Its fruits have high nutritional, health, and medicinal values. However, the development and ripening process of hawthorns is accompanied by dramatic changes in flavor, aroma, and bioactive phytochemicals, which are the fundamental factors that contribute to the potential health benefits and establishment of fruit quality. Therefore, an exploration of the dynamic changes in metabolites and their regulatory networks during the development of hawthorn fruits can elucidate the formation mechanisms of active substances in hawthorn fruits. In this study, we used a broad targeted metabolomics approach to identify and analyze the dynamics of metabolites in hawthorn fruits at five developmental stages. The results revealed 998 primary and secondary metabolites that were classified into 15 categories. The accumulation levels of most sugars increased during fruit development and then accelerated at the fruit ripening stage. The accumulation levels of a few organic acids (e.g., citric acid, isocitric acid, and quinic acid) continuously increased. Many organic acids exhibited significant decreasing trends. Among the 561 secondary metabolites detected, 189 were phenolic acids and 199 were flavonoids. The levels of many flavonoids were significantly reduced at later stages of fruit development; in contrast, the levels of two anthocyanins significantly increased during fruit ripening. Correlation analysis revealed that there is a certain correlation within and between primary as well as secondary metabolites during fruit development. Furthermore, the integration of metabolomic and transcriptomic data in this study revealed that changes in the expression of some differentially expressed genes (DEGs) were associated with the accumulation of metabolites such as sugars, organic acids, and flavonoids, e.g., the upregulated expression levels of *CS* (citrate synthase) genes were consistent with the continued accumulation of citric acid. Overall, this study demonstrates the metabolic changes that occur during the development of hawthorn fruit, explores the molecular mechanisms that underlie metabolite changes during fruit development, and lays a strong theoretical foundation for the improvement of hawthorn fruit quality and the development of functional components.

## 1. Introduction

Hawthorn (*Crataegus pinnatifida*) belongs to the genus *Crataegus* in the family *Rosaceae* [1]. *Crataegus pinnatifida* var. *major* is an endemic variety and an important cultivated fruit tree in China. Its fruit is one of the most widely consumed horticultural crops in China, either in fresh or processed form, due to its pleasant flavor, attractive color, and rich nutrition [2,3]. It is also an important raw material for functional foods and has been used as herbal medicine in the Chinese Pharmacopeia [4,5,6,7,8]. Currently, hawthorn has received considerable attention worldwide as a superior medicinal tonic because of its richness in biologically active substances [9,10]. However, hawthorns undergo a development process accompanied by dramatic changes in flavor, aroma, and bioactive phytochemicals, which are the fundamental factors behind their potential health benefits and establish their fruit quality. Accordingly, investigating the metabolic variations during hawthorn fruit development can provide a better understanding of the formation of bioactive phytochemicals and fruit quality.

Metabolomic studies, based on the qualitative and quantitative analysis of small-molecule metabolites, can be used to elucidate metabolic pathways or metabolic networks. Metabolomic analyses using ultra-high performance liquid chromatography-tandem mass spectrometry (UPLC-MS/MS) are important to food metabolomics because of their high sensitivity, specificity, and throughput [11,12]. Transcriptomic studies are based on the collection of all transcriptional products in an organism’s tissues under specific physiological conditions; such studies can be used to explore key regulatory mechanisms, metabolic processes, and other important traits [13,14,15]. The flavor of hawthorn fruit is the result of numerous organoleptic and volatile primary and secondary metabolites. More than 150 bioactive molecules have been identified in hawthorn, including phenolic acids, flavonoids, terpenoids, lignans, organic acids, and sugars [1]. Numerous studies have shown that the bioactive chemicals in hawthorn exhibit various functions, including anti-inflammatory, anti-viral, antioxidant, lipid-lowering, and blood sugar-regulating roles [4,16,17,18,19].

Most recent studies on hawthorn metabolites have focused on a single type of functional metabolites, such as lignans, flavonoids, tannins, etc. [20,21,22,23]; few studies have been conducted involving the broadly targeted metabolomic analysis of hawthorn. Although ripe hawthorn fruits contain numerous flavonoids with medicinal properties, their contents are comparatively lower than in young fruits. Additionally, the hawthorn is an important fruit with high yield and high nutritional value, but it is most often produced in a processed manner, rather than eaten fresh, because of its acidic taste. Therefore, research regarding the formation of metabolites in hawthorn fruit, with the intent of improving its medicinal value and enhancing its fresh taste, is a key focus of hawthorn research. Studies of metabolite accumulation during fruit development and ripening can provide insights regarding factors that underlie fruit quality [24]. Extensive and comprehensive metabolomic research is essential for an understanding of metabolite formation in hawthorn fruit.

In this study, we used an extensive targeted metabolomics approach based on ultra-high performance liquid chromatography-tandem mass spectrometry (UPLC-MS/MS) to identify and analyze the composition of metabolites and their changes during five periods of fruit development; ‘Shandong Dajinxing’ is one of the main varieties of Chinese hawthorn, with many excellent traits such as large fruit size, adaptability, and high yield. Therefore, we chose ‘Shandong Dajinxing’ as the test material to obtain insights into the metabolic basis of hawthorn fruit development. Metabolite-to-metabolite correlations were assessed to identify potential linkages between metabolites during hawthorn fruit development. An additional transcriptomic analysis of fruits at five developmental stages was conducted to investigate the transcriptional regulation and molecular mechanisms of metabolic changes during hawthorn fruit development. The findings are expected to provide a foundation for the in-depth research and development of hawthorn nutrients, functional components, and active ingredients.

## 2. Materials and Methods

### 2.1. Plant Materials

The cultivar ‘Shandong Dajinxing’, used as the test material, was cultivated at the hawthorn resource nursery of the Institute of Forestry and Pomology, Beijing Academy of Agriculture and Forestry Sciences. Three healthy fruit trees were selected in the same field and grown under standard cultivation conditions. Fruits were collected at 30, 60, 90, 120, and 150 days after full-bloom (DAF). A sample of 30 berries was randomly collected from each tree at each time point. The pulp, excluding the pits, was isolated in the laboratory, immediately frozen in liquid nitrogen for ≤5 min, and stored at −80 °C. Three biological replicates were used for subsequent broad-target metabolomic and transcriptomic analyses.

### 2.2. Broadly Targeted Metabolomic Analysis

Broadly targeted metabolomic analysis was performed by Metware Biotechnology Co., Ltd. (Wuhan, China) (http://www.metware.cn/ (accessed on 19 November 2021)).

Sample preparation and extraction

Fruit samples were dried in a vacuum freeze-dryer (Scientz-100F). Each freeze-dried sample was crushed using a mixer mill (MM 400, Retsch) with a zirconia bead for 1.5 min at 30 Hz. Then, 100 mg of lyophilized powder was dissolved in 1.2 mL of a 70% methanol solution, vortexed six times for 30 s each at an interval of 30 min, and placed in a refrigerator at 4 °C overnight. After centrifugation at 12,000 rpm for 10 min, the extracts were filtered (SCAA-104, 0.22-μm pore size; ANPEL, Shanghai, China, http://www.anpel.com.cn/ (accessed on 22 November 2021)) prior to UPLC-MS/MS analysis.

UPLC conditions

Sample extracts were analyzed using a UPLC-ESI-MS/MS system (UPLC, SHIMADZU Nexera X2, https://www.shimadzu.com.cn/ (accessed on 29 November 2021); MS, Applied Biosystems 4500 Q TRAP, https://www.thermofisher.cn/cn/zh/home/brands/applied-biosystems.html (accessed on 29 November 2021)). The following analytical conditions were used: the UPLC column was Agilent SB-C18 (1.8 μm, 2.1 mm × 100 mm). The mobile phase consisted of solvent A, pure water with 0.1% formic acid, and solvent B, acetonitrile with 0.1% formic acid. Sample measurements were performed using a gradient program with starting conditions of 95% A and 5% B. Within 9 min, a linear gradient to 5% A and 95% B was programmed; this composition was maintained for 1 min. Subsequently, a composition of 95% A and 5% B was reached within 1.1 min, then maintained for 2.9 min. The flow velocity was 0.35 mL per minute, the column oven temperature was 40 °C, and the injection volume was 4 μL. The effluent was connected to an ESI-triple quadrupole-linear ion trap (Q TRAP)-MS.

ESI-Q TRAP-MS/MS

Linear ion trap and triple quadrupole scans were conducted on a triple quadrupole-linear ion trap (Q TRAP) mass spectrometer, the AB4500 Q TRAP UPLC/MS/MS System, which was equipped with an ESI Turbo Ion-Spray interface; the mass spectrometer was operated in positive and negative ion mode and was controlled with Analyst 1.6.3 software (AB Sciex). The following ESI source operating parameters were used: ion source, turbo spray; source temperature, 550 °C; ion spray voltage, 5500 V (positive ion mode)/−4500 V (negative ion mode); ion source gas I, gas II, and curtain gas set at 50, 60, and 25.0 psi, respectively; and collision-activated dissociation, high. Instrument tuning and mass calibration were performed with 10 and 100 μmol/L polypropylene glycol solutions for the triple quadrupole and linear ion trap modes, respectively. Triple quadrupole scans were acquired as multiple reaction monitoring experiments with the collision gas (nitrogen) setting at medium. The declustering potential and collision energy for individual multiple reaction monitoring transitions were determined and optimized. A specific set of multiple reaction monitoring transitions was monitored for each period, according to the metabolites eluted within that period.

Metabolite characterization and quantification

Mass spectrometry data were processed using the software Analyst 1.6.3. Substance characterization was performed based on the secondary spectrum information, based on the Metware database (http://www.metware.cn/ (accessed on 1 December 2021)), with analysis removing isotopic signals, duplicate signals containing K+ ions, Na+ ions, NH4+ ions, and duplicate signals of fragment ions that are themselves other larger molecular weight substances.

Metabolite quantification was accomplished using triple quadrupole mass spectrometry in multiple reaction monitoring (MRM) mode. The characteristic ions of each substance were screened out through monitoring. The signal intensities of the characteristic ions were obtained in the detector and the peaks were integrated and calibrated using MultiaQuant software, with the peak area of each peak representing the relative content of the corresponding substance.

The total ion current (TIC) of the mixed QC samples is shown in Figure S1, and the MRM metabolite detection multi-peak graph is shown in Figure S2.

### 2.3. RNA Extraction and Transcriptome Analysis

The E.Z.N.A.^®^ Plant RNA Kit R6827 (Omega Bio-Tek, Norcross, GA, USA) was used to extract RNA from test samples. RNA degradation and contamination were monitored with 1% agarose gel. RNA purity was evaluated using the Nano Photometer^®^ spectrophotometer (IMPLEN, Westlake Village, CA, USA). RNA concentrations were measured using the Qubit^®^ RNA Assay Kit with the Qubit^®^2.0 Fluorometer (Life Technologies, San Mateo, CA, USA), and RNA integrity was assessed using the RNA Nano 6000 Assay Kit with the Bioanalyzer 2100 system (Agilent Technologies, Santa Clara, CA, USA).

In total, 1 μg of RNA per sample was used as input material for RNA sample preparation. Briefly, sequencing libraries were generated using the NEBNext^®^ UltraTM RNA Library Prep Kit for Illumina^®^ (New England Biolabs, Beverly, MA, USA), in accordance with the manufacturer’s instructions; index codes were added to facilitate sequence data association with each sample. Library fragments were purified with the AMPure XP system (Beckman Coulter, Miami, MA, USA), and library quality was assessed with the Agilent Bioanalyzer 2100 system. Clustering of index-coded samples was performed on the cBot Cluster Generation System using the TruSeq PE Cluster Kit v3-cBot-HS (Illumina), in accordance with the manufacturer’s instructions. After cluster generation, libraries were sequenced on the Illumina HiSeq platform, and 125-bp/150-bp paired-end reads were generated. Then, fastp v. 0.19.3 was used to filter the original data; all subsequent analyses were conducted using the resulting clean reads. Transcriptome assembly was performed using Trinity (v2.11.0). Trans Decoder (https://github.com/TransDecoder/TransDecoder/wiki (accessed on 20 December 2021)) was used to identify candidate coding regions within transcript sequences generated via de novo RNA-Seq transcript assembly using Trinity. Gene expression levels were estimated with RSEM software; the fragments per kilobase of transcript per million mapped reads (FPKM) value for each gene was calculated based on the gene length. DESeq2 v1.22.1 was used to analyze the differential expression between the two groups. *p*-values were corrected using the Benjamini–Hochberg method. Corrected *p*-values and |log_2_foldchange| values were used as thresholds for significant differential expression.

### 2.4. Data Processing and Statistical Analysis

Significant differences among samples were identified by analysis of variance using SPSS (version 26.0; SPSS Inc., Chicago, IL, USA); pairwise differences were explored using the Tukey test. *p*-values < 0.05 were considered statistically significant. Unsupervised principal component analysis was performed with the statistics function prcomp of R (www.r-project.org (accessed on 23 December 2021)). Significantly regulated metabolites between groups were determined based on variable importance in projection ≥ 1 and |log_2_foldchange| ≥ 1. Identified metabolites were annotated using the KEGG Compound Database (http://www.kegg.jp/kegg/compound/ (accessed on 27 December 2021)). Annotated metabolites were mapped to the KEGG Pathway Database (http://www.kegg.jp/kegg/pathway.html (accessed on 28 December 2021)).

## 3. Results

### 3.1. Dynamic Metabolic Profiles at Five Hawthorn Developmental Stages Revealed via UPLC-MS/MS Analysis

**Figure 1:**
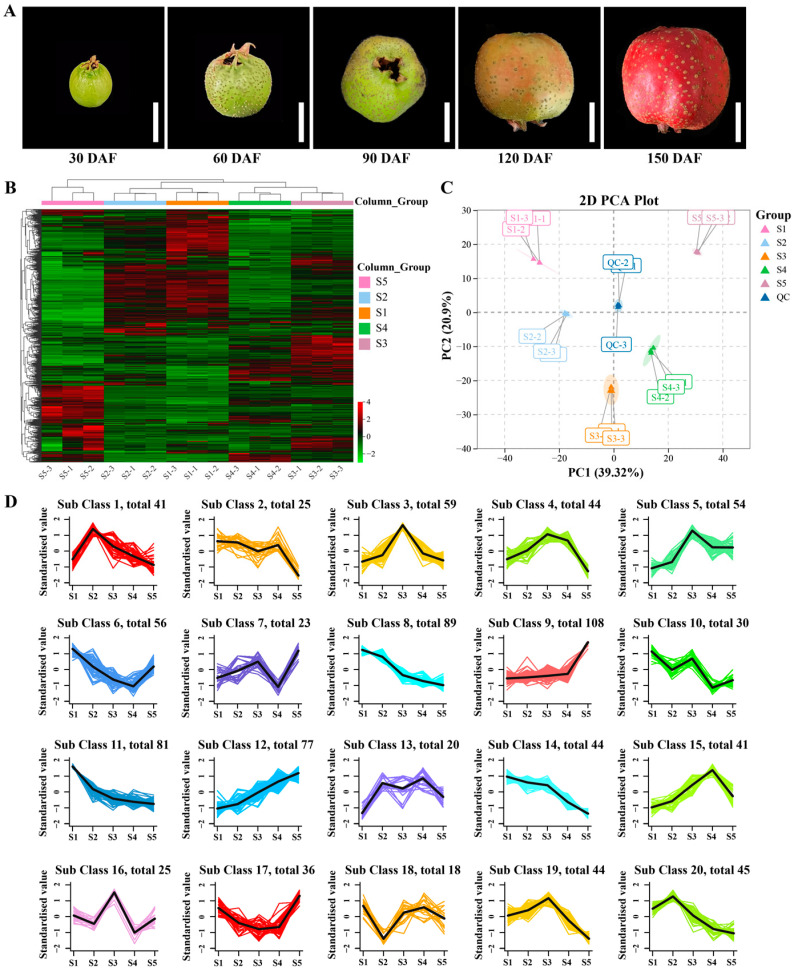
Dynamic metabolome of hawthorn fruit during development. (**A**) Fruit morphologies of ‘Shandong Dajinxing’ at five developmental stages; (**B**) heat map of all metabolites identified in hawthorn fruit at the five developmental stages; (**C**) principal component analysis of metabolomic data from hawthorn fruits at the five developmental stages; (**D**) cluster analysis of all 998 metabolites according to their concentration trends among the five fruit developmental stages. S1, S2, S3, S4, and S5 represent the five developmental stages of hawthorn fruit: S1, 30 days after full-bloom (DAF); S2, 60 DAF; S3, 90 DAF; S4, 120 DAF; and S5, 150 DAF.

To investigate the metabolic changes during hawthorn development, we used a metabolomics approach based on UPLC-MS/MS for a broadly targeted metabolic analysis of hawthorn fruits across five developmental stages. The fruit morphologies of ‘Shandong Dajinxing’ at the five tested developmental stages are shown in Figure 1A. After identification and analysis, 998 metabolites were detected (Table S1); the levels and trends of each metabolite are depicted in heat map format in Figure 1B. After identification of the overall metabolic differences between the test samples and the magnitude of variation between samples within the group, a principal component analysis of metabolites (including quality control samples) revealed significant differences in the metabolite profiles of fruit among the five developmental stages, along with a smaller degree of variation among the three replicate samples within each group (Figure 1C). Cluster analysis of the detected metabolites revealed that the 998 metabolites could be classified into 20 types based on their content trends, which are illustrated in Figure 1D.

### 3.2. Characteristics of Dynamic Changes in Primary Metabolites during Hawthorn Fruit Development

#### 3.2.1. Metabolism of Sugars and Alcohols

**Figure 2:**
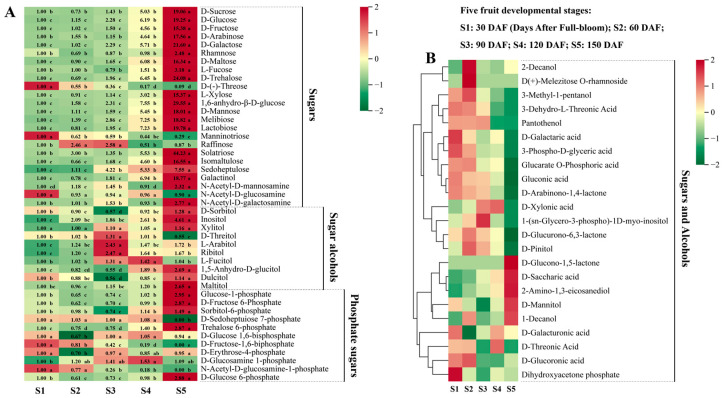
Sugar and alcohol metabolites detected at the five fruit developmental stages through broadly targeted UPLC-MS/MS. (**A**) Types and contents of sugars, sugar alcohols, and phosphate sugars in ‘Shandong Dajinxing’ fruits at the five developmental stages. S1, S2, S3, S4, and S5 represent the five fruit developmental stages of 30, 60, 90, 120, and 150 days after full-bloom (DAF), respectively. The first period of 30 DAF was used as a calibration value to calculate metabolite ploidy across the five periods. Significance analysis was performed using SPSS software with Tukey’s test and using a threshold of *p* < 0.05; the results are indicated by lowercase letters a, b, c, d, and e in the figure. Different letters indicate different levels of significance for comparisons between two samples. (**B**) Changes in the contents of other sugar and alcohol metabolites across the five periods of fruit development. Different colors on the heat map indicate the proportional contents of each metabolite, as determined based on the mean peak response area normalized to an *R* scale. Three independent biological replicates were assessed for each period.

In this study, 68 sugar and alcohol metabolites were detected in hawthorn fruits (Figure 2). Seven sugars, including D-glucose, D-fructose, D-galactose, D-mannose, melibiose, sedoheptulose, and 1,6-anhydro-β-D-glucose, exhibited continuous increases across the five stages of fruit development. The accumulation of D-sucrose, D-maltose, D-trehalose, lactobiose, isomaltulose, galactinol, rhamnose, and L-xylose exhibited non-significant decreases from the S1 stage to the S2 stage; D-arabinose, L-fucose, and solatriose all exhibited various extents of decrease from S2 to S3. However, all 11 of these sugars exhibited overall increasing trends across the five stages of fruit development. Notably, all 18 of these identified sugars significantly increased from S4 to S5; sucrose, glucose, and fructose, the main soluble sugars in the fruit, increased to levels that were 19.06-, 19.25-, and 15.38-fold higher, respectively, at S5 than at S1. The content of raffinose continued to increase in fruits from S1 to S3, significantly decreased from S3 to S4, and did not reach the S1 level from S4 to S5, although it partially increased again. Furthermore, N-acetyl-D-mannosamine and N-acetyl-D-galactosamine levels increased in the period of S1 to S3, significantly decreased from S3 to S4, and then significantly increased from S4 to S5, reaching a level more than twofold above the S1 level. No significant differences in N-acetyl-D-glucosamine content were observed among the five periods of fruit development. Additionally, the contents of D-(-)-threose and manninotriose exhibited consistent decreasing trends throughout the five stages of fruit development.

In total, 10 sugar alcohol metabolites were detected: D-sorbitol, dulcitol, xylitol, inositol, 1,5-anhydro-D-glucitol, D-threitol, maltitol, L-fucitol, ribitol, and L-arabitol. The levels of D-sorbitol, dulcitol, and 1,5-anhydro-D-glucitol all decreased from S1 to S3, then rapidly increased from S3 to S5. The content of xylitol did not significantly vary among the developmental stages. Finally, the contents of inositol, 1,5-anhydro-D-glucitol, and maltitol in the fruits at S5 increased to levels that were 4.61-, 2.69-, and 2.65-fold greater than their respective levels at S1.

Phosphate sugar metabolites play roles in sugar metabolism as important intermediates. The levels of glucose-1-phosphate, D-glucose 6-phosphate, and D-fructose 6-phosphate decreased from S1 to S2, gradually increased from S2 to S4, and then significantly increased from S4 to S5, such that they reached levels 2.95-, 2.88-, and 2.87-fold greater than their respective levels at S1. The contents of D-fructose-1,6-bisphosphate and N-acetyl-D-glucosamine-1-phosphate were undetectable at all five stages of fruit development. The trend of sorbitol-6-phosphate was identical to the trend of D-sorbitol. Trehalose 6-phosphate exhibited a decreasing and then increasing trend during fruit development, such that its level was 2.87-fold higher at S5 than at S1. No significant change in the content of D-sedoheptulose 7-phosphate was observed from S1 to S4, but it became undetectable at S5.

#### 3.2.2. Organic Acid Metabolism during Hawthorn Development

**Figure 3:**
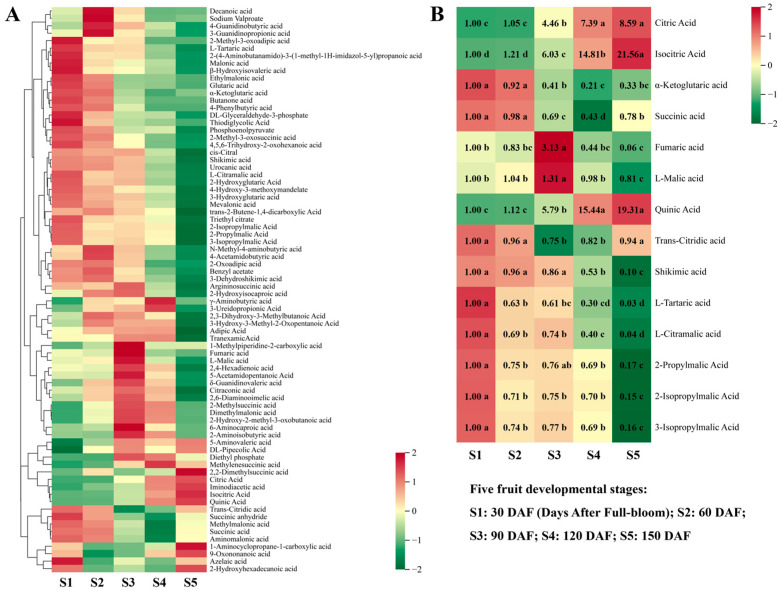
Changes in organic acid metabolism among the five developmental stages of fruit. (**A**) Heat map of the metabolism of 76 organic acids; (**B**) heat map and differential fold changes in the metabolism of 14 representative organic acids. The first period of 30 days after full-bloom (DAF) was used as a calibration value to calculate metabolite ploidy across the five periods. Significance analysis was performed using SPSS software with Tukey’s test and using a threshold of *p* < 0.05; the results are indicated by lowercase letters a, b, c, d, and e in the figure. Different letters indicate different levels of significance for comparisons between two samples. Different colors on the heat map indicate the proportional contents of each metabolite, as determined based on the mean peak response area normalized to an *R* scale. Three independent biological replicates were assessed for each period.

In total, 76 organic acid metabolites were detected in hawthorn fruit. With the exception of a few organic acids (e.g., citric acid and quinic acid) that exhibited increasing trends, the contents of most organic acids exhibited overall decreasing trends across the five stages of fruit development, with generally lower contents at S5 than at S1 (Figure 3A). The citric and isocitric acid contents continued to increase throughout fruit development, reaching their highest levels at S5, when they were 8.59- and 21.56-fold higher, respectively, than at S1 (Figure 3B). Notably, except for citric acid and isocitric acid, the contents of other intermediate organic acids involved in the tricarboxylic acid (TCA) cycle (α-ketoglutaric acid, succinic acid, fumaric acid, and L-malic acid) were all lower at S5 than at S1. The content of α-ketoglutaric acid and succinic acid gradually decreased from S1 to S4, and then increased from S4 to S5. The contents of fumaric acid and L-malic acid exhibited increasing and then decreasing trends, such that the content of fumaric acid at S5 was 0.06-fold of the content of S1, whereas the content of L-malic acid did not significantly change during fruit development. Additionally, we found that the trend and increase in quinic acid content were very similar to the trend and increase in isocitric acid, which was 19.31-fold higher at S5 than at S1.

#### 3.2.3. Accumulation Curves for Other Primary Metabolites

**Figure 4:**
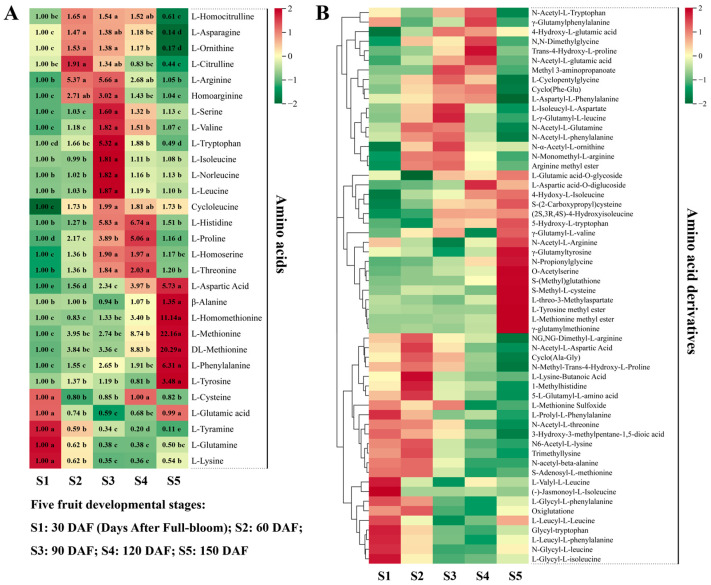
Amino acids (left) and their derivatives (right) detected through broadly targeted UPLC-MC/MS at the five fruit developmental stages. (**A**) Ploidy changes in each amino acid metabolite across the five periods of fruit development are depicted in heat map format; Significance analysis was performed using SPSS software with Tukey’s test and using a threshold of *p* < 0.05, the results are indicated by lowercase letters a, b, c, d, and e in the figure. (**B**) heat map of the metabolism of amino acid derivatives.

Overall, 29 amino acids and 58 amino acid derivatives were detected in hawthorn fruit; the changes in amino acids and their derivatives during fruit development are shown in Figure 4. Amino acids that exhibited high accumulation levels at maturity (period S5) were L-aspartic acid (Asp), β-alanine (Ala), L-homomethionine, L-methionine (Met), DL-methionine (DL-Met), L-phenylalanine (Phe), and L-tyrosine (Tyr). Conversely, amino acids that exhibited high accumulation levels during the early stages of fruit development were L-cysteine (Cys), L-glutamic acid (Glu), L-tyramine, L-glutamine (Gln), and L-lysine (Lys). All other amino acids increased and then decreased during fruit development; the maximum levels of L-homocitrulline, L-asparagine (Asn), L-ornithine, and L-citrulline were reached during the S2 period (60 DAF), after which they gradually decreased. The accumulation levels of L-arginine (Arg), homoarginine, L-serine (Ser), L-valine (Val), L-tryptophan (Trp), L-isoleucine (Ilu), L-norleucine, L-leucine (Leu), and cycloleucine were highest in the fruit at S3 (90 DAF); afterward, they tended to decrease. The levels of L-histidine (His), L-proline (Pro), L-homoserine, and L-threonine (Thr) increased from S1 to S4, peaked at S4, and decreased from S4 to S5.

In total, 49 nucleotides and their derivatives were detected in hawthorn fruit (Figure S3). Approximately half of these nucleotides and derivatives (e.g., uracil, adenine, guanine, guanosine, and xanthosine) accumulated at high levels in the early stages of fruit development, then gradually decreased in content as the fruit developed. A high accumulation of a few nucleotide derivatives was observed at fruit ripening. The contents of approximately one-fifth of nucleotides and their derivatives (e.g., ribosyladenosine, inosine, and xanthine) increased and then decreased during fruit development. A few other nucleotides and derivatives exhibited S-shaped accumulation curves during fruit development.

In total, 18 different vitamins were detected in hawthorn fruit (Figure S3), including L-ascorbic acid (VC) and dehydroascorbic acid; vitamins B1, B2, B3, B5, B6, B7, and their derivatives; and vitamins K1 and K2. The levels of most of these vitamins were significantly lower at fruit ripening (period S5) than at the beginning of fruit development. The content of VC tended to increase and then decrease, with the highest content in fruit at S2.

In total, 139 lipid metabolites were detected (Figure S4), including 75 free fatty acids, 1 phosphatidylcholine (PC), 26 lysophosphatidylcholine (LPC), 18 lysophosphatidylethanolamine (LPE), 15 glycerol ester, and 4 sphingolipids. In total, 23 kinds of free fatty acids (including γ-linolenic acid and α-linolenic acid), 4 kinds of lysophosphatidylcholines (including lysoPC 16:0), most glycerol esters, and all 4 sphingolipids were highly accumulated during fruit ripening.

### 3.3. Accumulation of Secondary Metabolites during Hawthorn Fruit Development

#### 3.3.1. Metabolism of Phenolic Acid Compounds

**Figure 5:**
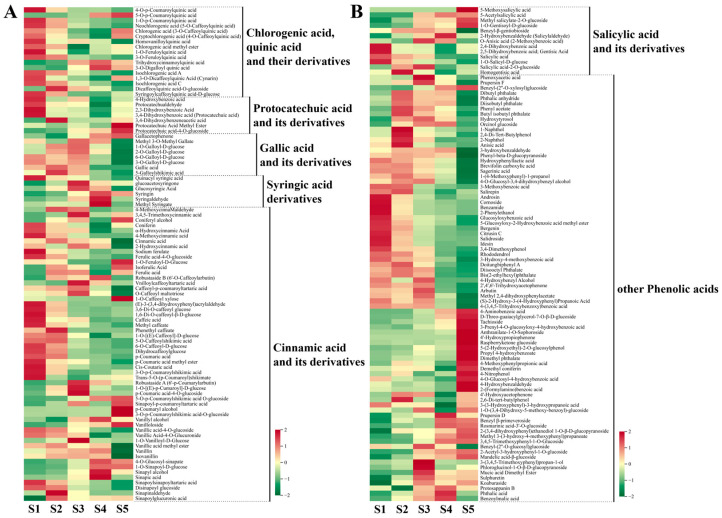
Cumulative distributions of phenolic acid compounds detected through broadly targeted UPLC-MC/MS at the five fruit developmental stages. (**A**) Metabolic heat map of chlorogenic/quinic acid and its derivatives, protocatechuic acid and its derivatives, gallic acid and its derivatives, syringic acid derivatives, and cinnamic acid and its derivatives; (**B**) metabolic heat map of salicylic acid and its derivatives, along with other phenolic acids.

**Figure 6:**
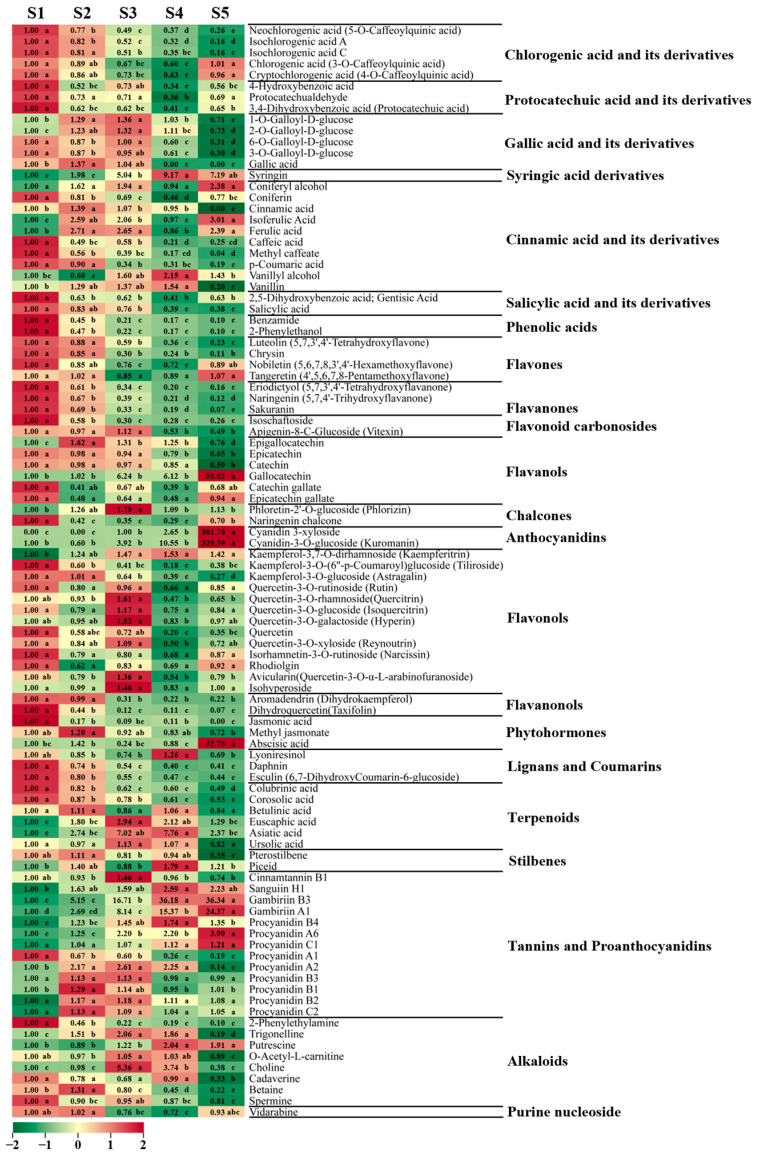
Representative secondary metabolites detected through broadly targeted metabolomic analysis during the five periods of fruit development. Ploidy changes for each metabolite across these five periods are depicted in heat map format. Significance analysis was performed using SPSS software with Tukey’s test and using a threshold of *p* < 0.05, the results are indicated by lowercase letters a, b, c, d, and e in the figure.

In this study, 189 phenolic acids were detected in hawthorn fruit (Figure 5). These 189 phenolic acids were subdivided into seven main groups: chlorogenic/quinic acid and its derivatives, protocatechuic acid and its derivatives, gallic acid and its derivatives, syringic acid derivatives, cinnamic acid and its derivatives, salicylic acid and its derivatives, and other phenolic acids (mostly benzoic acid derivatives). Changes in the contents of these groups during fruit development are shown in Figure 5. Most quinic acid derivatives accumulated at high levels in the early stages of fruit development, whereas chlorogenic acid and its derivatives gradually decreased in content as the fruit developed; however, the contents of chlorogenic acid and cryptochlorogenic acid returned to S1-period levels at fruit ripening (Figure 5A and Figure 6). Protocatechuic acid, 2,3-dihydroxybenzoic acid, protocatechualdehyde, and 4-hydroxybenzoic acid accumulated at high levels in the early stages of fruit development; the content of protocatechuic acid-4-O-glucoside gradually increased during fruit development. The contents of most gallic acids and their derivatives varied in an S-shaped curve throughout fruit development, with the highest level of gallic acid observed during the S2 period. The contents of the five syringic acid derivatives, with the exception of quinacyl syringic acid, first increased and then decreased during fruit development; the highest content of syringin was observed at S4 (the ripening stage) when it was 7.19-fold higher than the content at S1 (Figure 5A and Figure 6). Cinnamic acid and its derivatives (e.g., cinnamic acid, ferulic acid, caffeic acid, coumaric acid, vanillic acid, sinapic acid, and their derivatives), comprising 56 compounds, exhibited distinct trends across the five periods of fruit development. Additionally, phenolic acids such as salicylic acid, gentianic acid, and benzamide were highly accumulated in the early stages of fruit development; 4-aminobenzoic acid, 4-nitrophenol, and 4-hydroxybenzaldehyde were highly accumulated during fruit ripening (Figure 5B).

#### 3.3.2. Metabolism of Flavonoids

**Figure 7:**
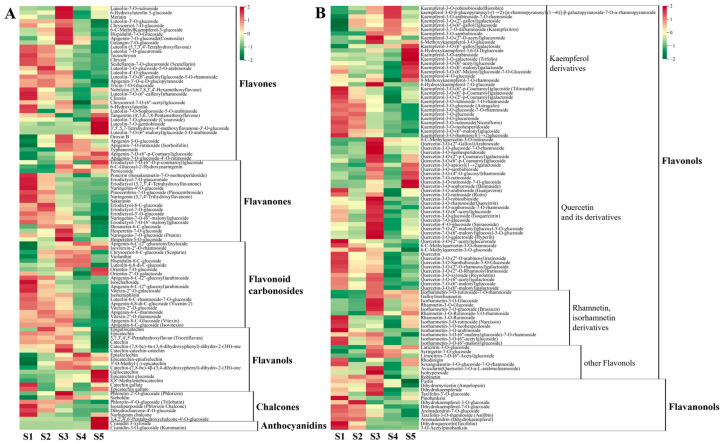
Cumulative distributions of flavonoids detected through broadly targeted UPLC-MC/MS at the five fruit developmental stages. (**A**) Metabolic heat map of flavonoids, flavanones, flavonoid carbonosides, flavanols, chalcones, and anthocyanidins; (**B**) metabolic heat map of flavonols and flavanonols.

In total, 199 flavonoid compounds were detected in hawthorn fruit (Figure 7), including flavonoids, flavanones, flavonoid carbonosides, flavanols, chalcones, anthocyanidins, flavonols, and flavanonol metabolites. Compared with the S1 period, most flavonoid levels decreased during the S2 period and a few increased during the S3 period; subsequently, significant reductions in numerous flavonoids were observed during the S4 and S5 stages (Figure 6 and Figure 7), indicating the inhibition of flavonoid biosynthesis during fruit ripening.

**Flavonoids**

In total, 36 metabolites were classified as flavonoids. Among them, flavonoid metabolites such as luteolin, chrysin, and clitorin exhibited high accumulation at the beginning of fruit development; 9 flavonoids (e.g., 6-hydroxyluteolin 5-glucoside, meratin, and luteolin-3′-O-glucoside) were at their highest levels during period S3 (90 DAF); and 7 flavonoids, including tangeretin and cynaroside, were at their highest levels during fruit ripening.

**Flavanones**

A total of 19 metabolites were classified as flavanones, including 7 eriodictyol and eriodictyol derivatives. Most flavanones (e.g., poncirin, eriodictyol, naringenin, and sakuranin) exhibited high accumulation at the beginning of fruit development, followed by decreasing contents during fruit development. Hesperetin-7-O-glucoside, naringenin-7-O-glucoside (prunin), and hesperetin-5-O-glucoside exhibited their highest levels at S3.

**Flavonoid carbonosides**

In total, 20 metabolites were classified as flavonoid carbonosides, including 2 luteolin glycosides, 7 apigenin glycosides, and 4 vitexin glycosides. More than half of the flavonoid carbonosides exhibited high accumulation only during the early stages of fruit development.

**Flavanols**

A total of 15 metabolites belonged to the flavanol group of substances. Among these substances, gallocatechin was 58.82-fold more abundant during S5 than during S1 (Figure 6). Additionally, catechin and catechin derivatives were less abundant during fruit ripening than at the beginning of fruit development.

**Chalcones**

In total, seven metabolites were assigned to the chalcone category. The contents of phlorizin and sieboldin increased and then decreased during fruit development, reaching their maximum values at S3. The levels of phlorizin chalcone and phloretin-4′-O-glucoside (trilobatin) gradually decreased during fruit development. Finally, the content of naringenin chalcone was highest at the beginning of fruit development; afterward, it gradually decreased before a slight increase in content at fruit ripening.

**Anthocyanidins**

The two anthocyanidin substances detected in hawthorn fruit were cyanidin 3-xyloside and cyanidin-3-O-glucoside (kuromanin), the contents of which significantly increased at fruit ripening, reaching levels more than 300-fold greater than their levels during S1 (Figure 6).

**Flavonols**

There were 88 metabolites classified as flavonols, including 31 kaempferol derivatives, 36 quercetin and derivatives, 13 rhamnetin and isorhamnetin derivatives, and 8 other flavonols. The content of kaempferitrin gradually increased during fruit development, then decreased during fruit ripening. In total, 9 kaempferol glycosides significantly increased during fruit ripening, whereas 12 kaempferol glycosides (e.g., tiliroside, astragalin, and nicotiflorin) were present at higher levels in the early stages of fruit development than in the late stages. Most flavonol metabolites (e.g., quercitrin, isoquercitrin, spiraeoside, hyperin, and isohyperoside) first increased and then decreased in content as the fruit developed; their highest contents were observed at S3. Additionally, the levels of rutin, quercetin, narcissin, and rhodiolgin in the fruit were all highest at 30 DAF.

**Flavanonols**

In total, 12 metabolites were classified as flavanonols. The highest levels of aromadendrin (dihydrokaempferol) and taxifolin (dihydroquercetin) were observed in the early stages of fruit development, which gradually decreased as the fruit developed.

#### 3.3.3. Accumulation of Other Secondary Metabolites (Excluding Flavonoids and Phenolic Acids)

In addition to phenolic acids and flavonoids, 173 other secondary metabolites were present in hawthorn, including phytohormones, lignans and coumarins, terpenoids, stilbenes, tannins, and alkaloids (Figure S5). The contents of numerous secondary metabolites significantly decreased during fruit ripening, indicating that the biosynthesis of many secondary metabolites was inhibited at that stage (Figure 5, Figure 6, Figure 7 and Figure S5).

**Phytohormones**

Three common phytohormones were detected: jasmonic acid, methyl jasmonate, and abscisic acid. Jasmonic acid exhibited high accumulation only at the early stages of fruit development, whereas the level of abscisic acid was significantly higher during fruit ripening than in the other four periods (32.76-fold higher than during S1) (Figure 6).

**Lignans and coumarins**

In total, 24 metabolites were classified as lignans or coumarins, including 17 lignans and 7 coumarins. The highest levels of lyoniresinol were observed during period S4, whereas the highest levels of daphnin and esculin were observed in the early stages of fruit development.

**Terpenoids**

There were 59 metabolites classified as terpenoids, including 57 triterpenes and 2 triterpene saponin metabolites. Among these compounds, 12 triterpenoids (e.g., colubrinic acid, corosolic acid, and maslinic acid) exhibited high accumulation at the beginning of fruit development, followed by decreasing contents as the fruit developed. Approximately half of all terpene metabolites exhibited their highest contents during S3, including the representative compounds euscaphic acid and ursolic acid.

**Stilbenes**

Two stilbenes were detected in hawthorn fruit: pterostilbene and piceid. The content of pterostilbene minimally varied from 30 DAF to 120 DAF but significantly decreased in the fruit at maturity. Piceid was most abundant in the fruit at 120 DAF.

**Tannins**

There were 14 kinds of tannin metabolites detected in hawthorn fruit, including 10 proanthocyanidin metabolites and 4 other tannins. Cinnamtannin B1 was most abundant in fruits during the S3 period. Gambiriin B3, gambiriin A1, procyanidin A6, and procyanidin C1 contents increased as the fruit developed; they were highest in the fruit at maturity, such that gambiriin B3 and gambiriin A1 were 36.34-fold and 24.37-fold more abundant in fruit at maturity than in the S1 period, respectively (Figure 6). Procyanidin A1 content decreased as the fruit developed. The levels of procyanidins A2, B3, B1, B2, and C2 increased and then decreased during fruit development.

**Alkaloids and other nitrogen-containing metabolites**

In total, 50 metabolites were classified as alkaloids, including 1 benzylphenylethylamine alkaloid, 1 quinoline alkaloid, 2 piperidine alkaloids, 2 pyridine alkaloids, 8 plumeranes, 13 phenolamines, and 23 other alkaloids. The levels of 2-phenylethylamine gradually decreased as the fruit developed. The levels of trigonelline and betaine increased and then decreased with fruit development; the trigonelline content was highest at S3 (2.04-fold higher than at S1), whereas the betaine content was highest at S2. The highest levels of putrescine were observed at S4 (2.04-fold higher than at S1) and the choline level was highest at S3 (5.36-fold higher than at S1). Amines such as cadaverine, spermine, and phenylethanolamine exhibited high accumulation levels in the early stages of fruit development.

### 3.4. Metabolite-Metabolite Correlations during Hawthorn Development and Ripening

**Figure 8:**
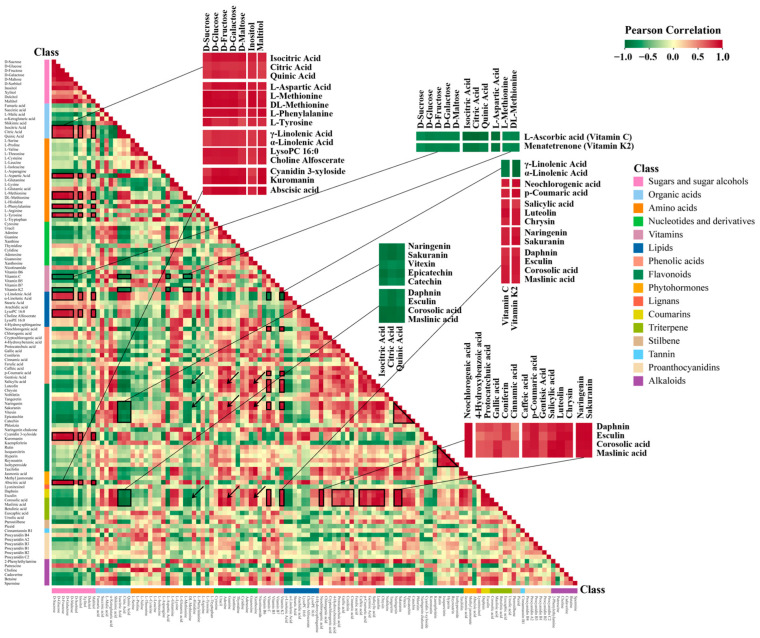
Heat map of correlations between metabolites. For this correlation analysis, 120 representative metabolites were selected from among 998 metabolites. The Pearson algorithm in R software was used to obtain metabolite–metabolite correlation coefficients. Each square of the heat map represents the correlation coefficient score derived from the Pearson analysis between metabolites located in the corresponding row and column. The 120 representative metabolites used for correlation analysis are listed in Table S2, and the correlation coefficient scores between metabolites are shown in Table S3. Information on correlations between some of the important metabolites is marked by black boxes and enlarged in the figure. Significant correlations between amino acids, nucleotides, and secondary metabolites are marked by black arrows.

To investigate the associations among metabolites during hawthorn fruit development, Pearson correlation coefficients were calculated for 120 representative metabolites. The heat map presented in Figure 8 shows correlations among those metabolites. Sugar and sugar alcohol metabolites (e.g., D-sucrose, D-glucose, D-fructose, D-galactose, D-maltose, maltitol, and inositol) were significantly negatively correlated with large numbers of primary and secondary metabolites; they were significantly positively correlated with a few primary and secondary metabolites, including three organic acids (citric acid, isocitric acid, and quinic acid; shown enlarged), five amino acids (L-aspartic acid, L-methionine, DL-methionine, L-phenylalanine, and L-tyrosine), four lipids, two anthocyanidins (shown enlarged), and abscisic acid. D-sorbitol and dulcitol had similar correlations with other metabolites, including correlations with most amino acids and a few other metabolites (e.g., secondary metabolites). Xylitol was only correlated with individual metabolites. Significant or highly significant positive correlations were observed between most sugar and sugar alcohol metabolites, with the exception of xylitol.

Regarding organic acids, we found highly significant positive correlations among citric, isocitric, and quinic acids; all three of these organic acids were significantly or highly significantly negatively correlated with large numbers of primary and secondary metabolites, including nucleotides, vitamins, phenolic acids, alkaloids, flavonoids, coumarins, and terpenoids (shown enlarged). Succinic acid, α-ketoglutaric acid, and shikimic acid exhibited significant positive correlations with most primary and secondary metabolites, in contrast to the correlations of citric acid, isocitric, acid, and quinic acid. L-malic acid had a highly significant positive correlation with fumaric acid and exhibited similar correlations with other metabolites.

Additionally, four amino acids (L-aspartic acid, L-methionine, DL-methionine, and L-phenylalanine) exhibited negative correlations with many secondary metabolites (indicated by arrows in Figure 8). Some amino acids (e.g., L-cysteine, L-leucine, L-isoleucine, L-arginine, and L-tryptophan) exhibited less significant correlations with other primary and secondary metabolites.

Excluding thymidine, nucleotides exhibited positive correlations with a broad range of secondary metabolites, including phenolic acids, most flavonoids except anthocyanidins, jasmonic acid, and methyl jasmonate, coumarins, some terpenoids, and alkaloids (indicated by arrows in Figure 8).

Lipids exhibited significant positive correlations with each other. Specifically, highly significant positive correlations were observed for γ-linolenic acid with α-linolenic acid (lipid combination 1), lysoPC 16:0 with choline alfoscerate (lipid combination 2), and lysoPE 16:0 with 4-hydroxysphinganine (lipid combination 3). Additionally, γ-linolenic acid, α-linolenic acid, lysoPC 16:0, and choline alfoscerate were positively correlated with sugars, organic acids, and some amino acids; they were negatively correlated with most nucleotides and vitamins, and significantly negatively correlated with some secondary metabolites (shown enlarged).

VC was negatively correlated with sugars, lipids, some organic acids, and amino acids; positively correlated with some secondary metabolites; and not significantly correlated with tannins. In contrast, menatetrenone (vitamin K2) was highly significantly correlated with VC (shown enlarged).

Phenolic acids exhibited obvious positive correlations with each other and mostly negative correlations with primary metabolites such as sugars, amino acids, some organic acids, and lipids; they had positive correlations with lots of secondary metabolites (e.g., most flavonoids excluding anthocyanidins, coumarins, corosolic acid, maslinic acid (shown enlarged), and some alkaloids).

For flavonoids, significant positive correlations were observed between luteolin and chrysin (flavonoids), naringenin and sakuranin (flavanones), vitexin (flavonoid carbonosides), and epicatechin and catechin (flavanols); highly significant positive correlations were observed among rutin, isoquercitrin, hyperin, isohyperoside, and reynoutrin (flavonols) (black triangular box in Figure 8). Anthocyanidins were significantly correlated with various metabolites, such that significant or highly significant positive correlations were observed for nine sugars, three organic acids, six amino acids, and five lipids; negative correlations were observed for four nucleotides, four vitamins excluding nicotinamide, five phenolic acids including cinnamic acid, two flavonoids, two flavanones, one flavonoid carbonoside, two flavanols (catechin and epicatechin), two triterpenes (corosolic acid and maslinic acid), one stilbene (pterostilbene), procyanidin A2, and three amines (cadaverine, spermine, and betaine). Additionally, anthocyanidins exhibited a highly significant positive correlation with abscisic acid.

Among hormone metabolites, abscisic acid exhibited highly significant positive correlations with most sugar and sugar alcohol metabolites, five amino acids, two lipids, and anthocyanidins; it exhibited highly significant negative correlations with shikimic acid, L-asparagine, cinnamic acid, catechin, pterostilbene, and procyanidin A2. Jasmonic acid exhibited highly significant positive correlations with L-glutamine, L-lysine, chlorogenic acid, caffeic acid, naringenin, sakuranin, taxifolin, daphnin, esculin, and 2-phenylethylamine.

### 3.5. Differential Accumulation of Metabolites among Stages of Fruit Development

**Figure 9:**
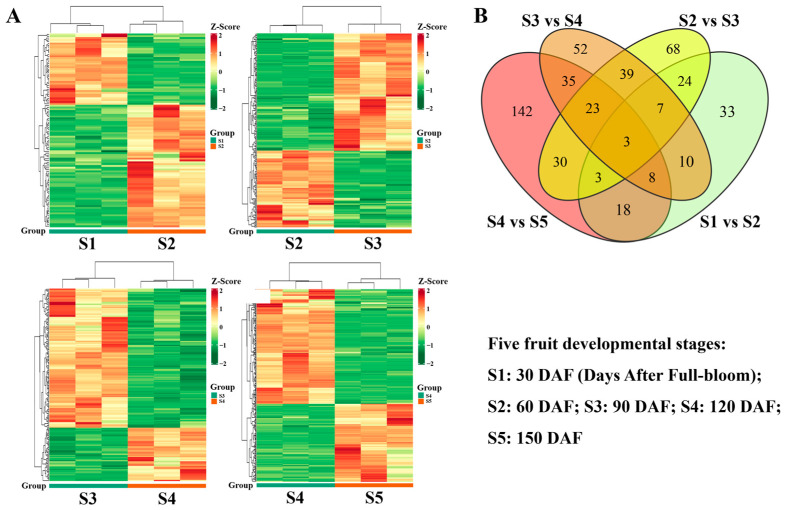
Differential accumulation of metabolites among the five stages of fruit development. (**A**) Heat map of differential metabolites between periods S1 and S2, S2 and S3, S3 and S4, and S4 and S5 (three independent replicates of each phase are shown); (**B**) Venn diagrams of differential metabolites between periods S1 and S2, S2 and S3, S3 and S4, and S4 and S5.

To investigate the changes in metabolites among stages of hawthorn fruit development, differential metabolites were analyzed between periods S1 and S2, S2 and S3, S3 and S4, and S4 and S5 (Figure 9). The significantly differential metabolites identified in these four comparisons are listed in Table S4. The results of the statistics for metabolites with significant differences at different stages are shown in Table 1. Volcano plots of differential metabolites at different stages are shown in Figure S6.

In total, 106 significantly differential metabolites were identified between S1 and S2, including 67 upregulated and 39 downregulated metabolites (Table 1, Figure S6), indicating that more differential metabolites were upregulated (rather than downregulated) during the early fruit expansion stage. In the comparison of S2 versus S3, 197 significantly differential metabolites were detected (119 were upregulated and 78 were downregulated), representing a significant increase in the number of differential metabolites and indicating that numerous metabolites accumulated in the fruit as it developed. In contrast, the number of differential metabolites between S3 and S4 was slightly lower than in the previous stage; it included more downregulated metabolites (S3 vs. S4, 177 significantly differential metabolites: 49 upregulated and 128 downregulated), suggesting that many differential metabolites were involved in the transition from the early fruit development expansion stage (S3) to the early fruit ripening stage (S4). Furthermore, the number of differential metabolites significantly increased between S4 and S5 (S4 vs. S5, 262 significantly differential metabolites: 106 upregulated and 156 downregulated), indicating that many metabolites accumulated in the fruit during the ripening stage (S5) (Table 1, Figure S6). The heat maps of differential metabolites in each of these four combinations clearly demonstrate these trends (Figure 9A). Additionally, the numbers of differential metabolites were compared among these four combinations using a Venn diagram (Figure 9B). As illustrated in Figure 9B, three groups of differential metabolites were associated with the four combinations. Overall, 37 differential metabolites were present between S1 vs. S2 and S2 vs. S3, 72 differential metabolites were present between S2 vs. S3 and S3 vs. S4, and 69 metabolites were present between S3 vs. S4 and S4 vs. S5.

To explore the biological processes associated with the differential metabolites, metabolites were assigned to KEGG pathways (Figure S7). As shown in Figure S7, the differential metabolites between S1 and S2 were significantly associated with ABC transporters, arginine and proline metabolism, butanoate metabolism, and purine metabolism (Table S5). Differential metabolites between S2 and S3 were significantly associated with the TCA cycle, linoleic acid metabolism, flavonoid biosynthesis, and phenylpropanoid biosynthesis. Differential metabolites between S3 and S4 were significantly associated with galactose metabolism, flavone and flavonol biosynthesis, and tryptophan metabolism. Differential metabolites between S4 and S5 were significantly associated with metabolic pathways such as starch and sucrose metabolism, 2-oxocarboxylic acid metabolism, nicotinate, and nicotinamide metabolism, biosynthesis of various secondary metabolites part 2 (biosynthesis of various antibiotics), pyruvate metabolism, and glycolysis/gluconeogenesis (Figure S7, Table S5). Additionally, the enrichment of carotenoid biosynthesis-related metabolites corresponds to the accumulation of abscisic acid in the fruit during the ripening stages (S3 to S5). Among all differential metabolites, 15 were mapped to the phenylpropanoid biosynthesis pathway, 12 were mapped to the flavonoid biosynthesis pathway, and 5 were mapped to the flavone and flavonol biosynthesis pathway (Table S5).

### 3.6. Expression Patterns of Genes Associated with Various Metabolic Pathways during Fruit Development and Ripening

To further investigate the transcriptional regulation of hawthorn fruit development and ripening, we sequenced the transcriptomes of fruits at the five developmental stages, then explored the potential associations between the metabolome and transcriptome during hawthorn development by assigning the differentially expressed genes (DEGs) between fruit developmental stages to various metabolic pathways.

**Figure 10:**
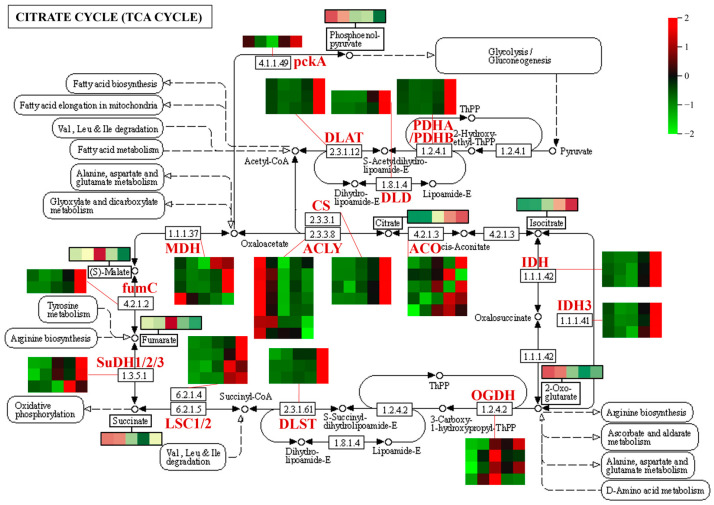
Expression patterns of differentially expressed genes (DEGs) associated with organic acid metabolism (TCA cycle) during hawthorn development. Enzyme classes in the metabolic pathway are indicated in red. Heat maps represent the expression patterns of DEGs involved in the metabolic pathway. On each heat map, rows represent different transcripts, and the five columns from left to right represent the five developmental periods (S1, S2, S3, S4, and S5: 30, 60, 90, 120, and 150 days after full-bloom, respectively). Changes in the contents of some metabolites (indicated by black boxes) during the five developmental periods of the fruit are indicated. MDH, malate dehydrogenase; CS, citrate synthase; ACLY, ATP citrate (pro-S)-lyase; ACO, aconitate hydratase; IDH1, isocitrate dehydrogenase; IDH3, isocitrate dehydrogenase (NAD+); OGDH, 2-oxoglutarate dehydrogenase E1 component; DLST, dihydrolipoamide succinyl transferase; LSC1, succinyl-CoA synthetase α subunit; LSC2, succinyl-CoA synthetase β subunit; SuDH1, succinate dehydrogenase (ubiquinone) flavoprotein subunit; SuDH2, succinate dehydrogenase (ubiquinone) iron-sulfur subunit; SuDH3, succinate dehydrogenase (ubiquinone) cytochrome b560 subunit; fumC, fumarate hydratase, class II; DLD, dihydrolipoamide dehydrogenase; pckA, phosphoenolpyruvate carboxykinase (ATP); DLAT, pyruvate dehydrogenase E2 component (dihydrolipoamide acetyltransferase); PDHA, pyruvate dehydrogenase E1 component α subunit; and PDHB, pyruvate dehydrogenase E1 component β subunit.

The expression patterns of DEGs involved in the TCA cycle metabolic pathway are shown in Figure 10. Many genes involved in the TCA cycle were upregulated during fruit development, however, the levels of intermediates in the TCA cycle (e.g., α-ketoglutarate, fumaric acid, citric acid, and isocitric acid) were reduced during fruit development, suggesting that the genes involved in the TCA cycle were activated but the corresponding metabolites were consumed as substrates for downstream metabolic pathways; the upregulated expression level of *CS* (citrate synthase gene) corresponded to the accumulation of citric acid during fruit development. A large number of genes involved in glycolysis/gluconeogenesis were also upregulated during fruit development (Figure S8), while numerous sugar metabolites (e.g., fructose, glucose, and sucrose) exhibited upward trends across the five periods of fruit development (Figure 2), suggesting that genes involved in glycolysis/gluconeogenesis were activated and the corresponding metabolites began to accumulate. Additionally, phosphoenolpyruvate is a key compound involved in many metabolic processes including glycolysis/gluconeogenesis, the TCA cycle, and pyruvate metabolism. The phosphoenolpyruvate content was highest in hawthorn fruits at the early stage of development, then gradually decreased. The accumulation pattern of phosphoenolpyruvate was negatively correlated with the expression level of *ENO* (enolase gene), which increased during fruit development and reached its maximum expression level at fruit ripening (Figure S8).

**Figure 11:**
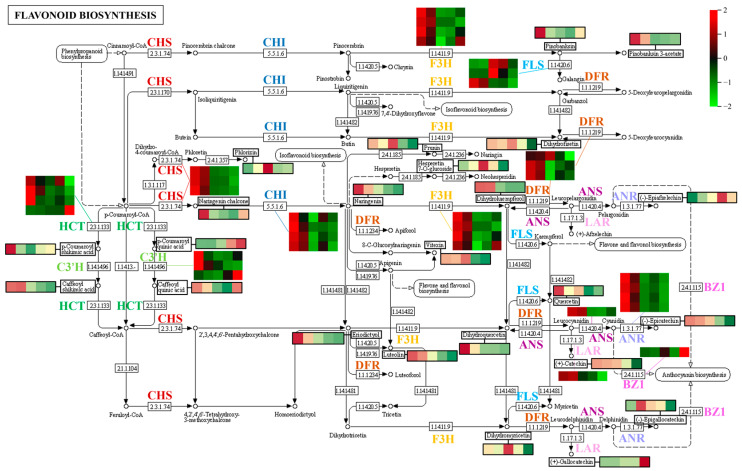
Expression patterns of differentially expressed genes (DEGs) associated with the flavonoid biosynthetic pathway during hawthorn development. Different enzymes in the flavonoid biosynthetic pathway are indicated by different colors. Heat maps in the figure represent the expression patterns of DEGs involved in the corresponding pathways. In each heat map, rows represent different transcripts; the five columns from left to right represent the five developmental periods (S1, S2, S3, S4, and S5: 30, 60, 90, 120, and 150 DAF, respectively). Changes in the contents of some metabolites (indicated by black boxes) across the five developmental periods of the fruit are also indicated in the figure. CHS, chalcone synthase; CHI, chalcone isomerase; F3H, naringenin 3-dioxygenase; FLS, flavonol synthase; DFR, bifunctional dihydroflavonol 4-reductase; ANS, anthocyanidin synthase; LAR, leucoanthocyanidin reductase; ANR, anthocyanidin reductase; HCT, shikimate O-hydroxycinnamoyl transferase; C3′H, 5-O-(4-coumaroyl)-D-quinate 3′-monooxygenase; and BZ1, anthocyanidin 3-O-glucosyltransferase.

We subsequently investigated the expression patterns of DEGs assigned to the flavonoid pathway (Figure 11). Most DEGs involved in the flavonoid pathway were downregulated during fruit development, including *CHS* (chalcone synthase gene), *CHI* (chalcone isomerase gene), *F3H* (naringenin 3-dioxygenase gene), *LAR* (leucoanthocyanidin reductase gene), and *ANR* (anthocyanidin reductase gene). The accumulation of naringenin chalcone was highest at the beginning of fruit development and gradually decreased from 60 to 120 DAF; it was positively correlated with the expression level of *CHS*. Naringenin content was positively correlated with the expression level of *CHI* at early stages of fruit development (30 to 60 DAF), but the expression level of *CHI* was lowest in fruits at 90 DAF; the minimum naringenin content occurred in mature fruit at 150 DAF. Pinobanksin was positively correlated with the expression level of *F3H*, and dihydrokaempferol and dihydroquercetin were positively correlated with the expression levels of *F3H* and *ANS* (anthocyanidin synthase gene). The catechin content was positively correlated with the expression level of *LAR*, the epiafzelechin content was negatively correlated with the expression level of *ANR*, and the epicatechin content was positively correlated with the expression level of *ANR*. The accumulation of epigallocatechin was low in fruit at 30 DAF and increased to its highest value at 60 DAF; the change in content from 30 to 60 DAF was negatively correlated with the expression level of *ANR*, whereas the change in content from 90 to 150 DAF was positively correlated with the expression level of *ANR*. Taken together, these results suggest that the expression levels of most DEGs were significantly lower at S5 than at S4, whereas the metabolomic data demonstrated that levels of numerous flavonoid metabolites were significantly lower at fruit ripening; thus, genes involved in the flavonoid pathway were presumably repressed, and the synthesis of corresponding metabolites was inhibited. Notably, the expression level of *BZ1* (anthocyanidin 3-O-glucosyltransferase gene) was significantly upregulated during the fruit ripening stage (S4 to S5); similarly, two anthocyanidins (cyanidin 3-xyloside and cyanidin-3-O-glucoside) accumulated in large quantities in ripening hawthorn fruit (Figure 6 and Figure 7), indicating that the *BZ1* gene was activated and may be responsible for pigment accumulation during the fruit coloring stage.

Additionally, p-coumaroyl-CoA is involved in the formation of some phenolic acids and plays roles in various metabolic pathways (e.g., flavonoid biosynthesis and phenylpropanoid biosynthesis). As shown in Figure 11, the formation of chlorogenic acid is associated with *C3*′*H* (5-O-(4-coumaroyl)-D-quinate 3′-monooxygenase gene), and the amount of chlorogenic acid in hawthorn fruit is positively correlated with the expression level of the *C3*′*H* gene.

## 4. Discussion

UPLC-MS/MS enables the large-scale detection, along with the accurate characterization and quantification, of substances [25]. In recent years, UPLC-MS/MS-based metabolomic analysis has dominated metabolomic studies of nutrient composition and fruit quality [15,26,27,28]. Furthermore, explorations of metabolome and transcriptome associations have been shown to help elucidate the genetic basis of metabolic networks [29]. Zhu et al. [26] analyzed the metabolite profiles of hundreds of tomato genotypes, combining genomic and transcriptomic methods to assess how breeding altered the metabolome of the tomato fruit; the results provided insights into metabolome-assisted breeding. Xu et al. [15] used a broadly targeted metabolomics approach to study a wide range of metabolite changes during the development and ripening of ‘Pinova’ apples; they combined the results with transcriptomic analysis to provide molecular-level insights regarding the metabolic mechanisms that underlie important fruit quality traits in commercial apples. Hawthorn is an important fruit that can directly or indirectly provide nutrition, energy, and medicinal compounds to humans; its fruits synthesize numerous flavonoids, phenolic acids, sugars, organic acids, and other metabolic substances with diverse biological functions. Therefore, studies focused on the metabolomics of hawthorn fruit can facilitate the research on the medicinal properties ascribed to its metabolites while providing data to support improvements in fruit quality. In this study, UPLC-MS/MS was used to identify 998 metabolites in hawthorn fruits at five developmental stages. These metabolites were classified into 15 categories and their dynamics during fruit development and ripening are described in this paper. Our findings provide insights into the extensive and comprehensive metabolomic changes that occur during hawthorn fruit development; they also provide a basis for further exploration of the metabolic mechanisms that underlie the fruit quality traits associated with the medicinal and fresh food properties of hawthorn.

Hawthorn fruit exhibited 437 primary metabolites, which could be subdivided into six types: sugars and alcohols, organic acids, amino acids and their derivatives, nucleotides and their derivatives, vitamins, and lipids. Sugars and organic acids are important metabolites that determine the sweet and sour flavors of fruit [30,31,32]. The glucose and fructose contents in hawthorn exhibited continuous upward trends throughout the five stages of fruit development, whereas the accumulation of sucrose slightly decreased from S1 to S2 and continued to increase from S2 to S5. The accumulation of many sugars (e.g., sucrose, glucose, and fructose) accelerated from S4 to S5 (Figure 2). These results suggest that sugar accumulation, which drives the sweetness of hawthorn fruit, mainly occurs during the fruit ripening stage. Additionally, the sorbitol content of hawthorn decreased from S1 to S3 and increased from S3 to S5, such that it was 1.28-fold higher at S5 than at S1. Considering that hawthorn, apple, and pear belong to the Rosaceae family, we compared the accumulation patterns of sugars and organic acids in hawthorn fruits with those in apples and pears. The pattern of sugar accumulation in hawthorn fruit in this study differed from the pattern in apples [15]; trends in the contents of sucrose, glucose, and fructose in apple fruits are similar, such that they rapidly increase in the early stages of fruit development, then slightly increase throughout the developmental period up to fruit ripening. The accumulation of sugars in apples is presumed to occur mainly at the early stages of fruit development; the sorbitol content of apples steadily changes during fruit development, then decreases during fruit ripening [15]. The fructose content of pears continuously increases from 1 to 5 months after flowering; the accumulation of glucose and sorbitol mainly occurs at the early stage of fruit development from 1 to 2 months after flowering [33]. Notably, the contents of citric, isocitric, and quinic acids in hawthorn continued to increase during fruit development, whereas the content of malic acid did not significantly vary among the stages of fruit development (Figure 3). This trend in organic acids considerably differs from the trends in apples and pears, which exhibit insignificant differences in the levels of citric acid accumulation between the periods of fruit development, whereas the levels of malic and quinic acids continue to decrease [15,33]. In conclusion, the contents of the main sugars and organic acids in hawthorn increase during fruit development; these differences in the accumulation of sugars and organic acids may be responsible for the lower sweetness of hawthorn fruit compared with apples and pears.

A typical feature of plants is their capacity to produce diverse secondary metabolites: compounds that are not directly involved in basic photosynthesis or respiratory metabolism but are considered essential for plant survival in the environment [34]. Thus, the abundance of secondary metabolite types in hawthorn may be responsible for its robust adaptability to environmental conditions. Phenolic acids, flavonoids, phytohormones, terpenoids, lignans, coumarins, stilbenes, alkaloids, and tannins were among the 561 secondary metabolites of the nine types detected and described in this study of hawthorn fruits. The rich variety and high contents of secondary metabolites are indicative of their medicinal value. Phenolic acids are considered to be one of the most important components of functional fruits, and most phenolic acids such as chlorogenic acid and gallic acid are believed to have free radical elimination, antioxidant, and anti-cancer effects [35,36]. Hawthorn fruit is rich in up to 189 phenolic acids, making it an excellent nutritional choice of healthy fruit. Flavonoids are an important class of secondary metabolites with diverse roles in plants, including resistance to environmental stresses [37]; numerous flavonoids have associations with human health [38,39]. Clarification of the regulatory mechanisms of flavonoid biosynthesis and enhancement of flavonoid contents are important topics in the study of secondary metabolites in fruits [40,41,42,43]. The findings of this study revealed that hawthorn fruits are rich in 199 flavonoids, a number that considerably exceeds the 104 flavonoids present in apples [15] and 117 flavonoids present in citrus species [44]. The first key rate-limiting enzyme in flavonoid biosynthesis is CHS [45], and, as we observed in Figure 11, naringin chalcone accumulation was positively correlated with *CHS* gene expression from 60 to 120 days after full-bloom. However, *CHS* gene expression continued to be downregulated, while naringin chalcone accumulation increased significantly at the fruit ripening stage from 120 to 150 days after full-bloom, at which point the accumulation of naringin chalcone was negatively correlated with the expression of the *CHS* gene, demonstrating that the function of *CHS* gene may be regulated by post-transcriptional modifications [45]. In this study, the expression of *CHS*, *F3H,* and *DFR* genes was highest in the pulp of ‘Shandong Dajinxing’ at the early stage of fruit development (30 days after full-bloom), this pattern of expression differs from the study by Zhao et al. [46]. The expression of *CHS*, *F3H,* and *DFR* genes in the flesh of ‘Xinglongzirou’ and ‘Bairangmian’ hawthorn increased and then decreased between 70 and 150 days after full-bloom, reaching a maximum value at 120 days after full-bloom. Many flavonoids in hawthorn are significantly reduced in abundance at fruit ripening (Figure 6 and Figure 7); this trend is associated with the downregulation of the expression levels of most DEGs in the flavonoid pathway. Moreover, the significant accumulation of anthocyanidins at the fruit ripening stage revealed a distinct association with the upregulated expression level of *BZ1* (anthocyanidin 3-O-glucosyltransferase gene).

## 5. Conclusions

This comprehensive exploration of metabolites and their variations during hawthorn fruit development provides insights into the metabolic basis of hawthorn fruit characteristics. Potential links within and between primary and secondary metabolites were identified through the assessment of metabolite-to-metabolite correlations. An in-depth exploration of the transcriptional regulation of metabolite changes was conducted through the integration of metabolomic and transcriptomic data. These data present a global view of metabolic changes during the developmental cycle of hawthorn fruit; they can help to elucidate the molecular regulation of sugar, organic acid, and flavonoid metabolism in hawthorn fruit. The findings of this study will be useful in clarifying the formation mechanisms of medicinal components and fruit qualities in hawthorn, providing a foundation for the development of medicinal properties and improvements in fruit quality.

## Figures and Tables

**Table 1:** Statistical table of significantly different metabolites.

Group Name	All	Down	Up
S1_vs_S2	106	39	67
S2_vs_S3	197	78	119
S3_vs_S4	177	128	49
S4_vs_S5	262	156	106

## Data Availability

All the data supporting the findings of this study are included in the present article and the supplementary files.

## Supplementary Materials

The following supporting information can be downloaded at: https://www.mdpi.com/article/10.3390/metabo13030423/s1, Figure S1: Total ion current (TIC) diagram of mixed QC samples; Figure S2: MRM (multiple reaction monitoring) metabolite assay multi-peak plots; Figure S3: nucleotides and their derivatives (A) and vitamins (B) detected by broadly targeted UPLC-MC/MS at five fruit developmental stages; Figure S4: lipids detected by broadly targeted UPLC-MC/MS at the five fruit developmental stages; Figure S5: secondary metabolites (excluding flavonoids and phenolic acids) detected by broadly targeted UPLC-MC/MS at the five fruit developmental stages; Figure S6:Volcano plots of differential metabolites between periods S1 and S2, S2 and S3, S3 and S4, and S4 and S5; Figure S7: KEGG pathway assignments of differential metabolites between periods S1 and S2, S2 and S3, S3 and S4, and S4 and S5; Figure S8: Expression patterns of differentially expressed genes (DEGs) involved in glycolysis/gluconeogenesis during hawthorn development; Table S1: the total 998 detected metabolites in this study; Table S2: the list of 120 representative metabolites for correlation analysis; Table S3: table of correlation coefficients between different representative metabolites; Table S4: differentially accumulated metabolites among S1 vs. S2, S2 vs. S3, S3 vs. S4, and S4 vs. S5, respectively; Table S5: KEGG pathway assignments of differentially accumulated metabolites among S1 vs. S2, S2 vs. S3, S3 vs. S4, and S4 vs. S5, respectively.
